# Reflections on the neural processing of echoes for distance information in echolocating bats

**DOI:** 10.1007/s00359-026-01804-6

**Published:** 2026-03-31

**Authors:** Alan D. Grinnell

**Affiliations:** Department of Physiology, UCLA, Los Angeles, CA 90095 USA

**Keywords:** Bats, Echolocation, Short CF-FM, Distance discrimination, Electrophysiology

## Abstract

Multiple neural specializations in echolocating bats enable target distance determination by measurement of the delay between many frequencies in the emitted FM signal and the same frequencies in returning echoes. In the neotropical lesser bulldog bat, *Noctilio albiventris*, a short constant frequency (CF) component preceding the FM sweep in emitted sounds is critical to the measurement of pulse-echo delay. The author reviews behavioral experiments that have a bearing on possible neural mechanisms for this role of the CF. Other experiments that substituted a descending sequence of brief pure tones for the normal FM sweep in evoking a normal approach phase behavioral response reveal interesting differences between *N. albiventris* and the purely FM bat *Eptesicus fusc*us in several parameters: the minimum number of pure tone frequencies necessary in the “FM sweep” for distance determination, the minimum effective frequency difference between steps, and the maximum duration of pure tone steps.

## Introduction

It is an honor to contribute an article in a journal issue dedicated to my close friend and colleague, Peter Narins. I apologize that it is about bats instead of frogs, but at least it does not focus on frog-eating bats. In this paper I review some of what we know about the different features of the frequency modulation (FM) sweep that are critical for distance determination in echolocation, and about the role of the brief constant frequency (CF) component preceding the FM sweep in “short CF-FM” bats-largely a mystery up to this point.. Although much of this information is well established, other aspects, in my opinion, deserve further consideration.

### The importance of echolocation call bandwidth

**Fig. 1:**
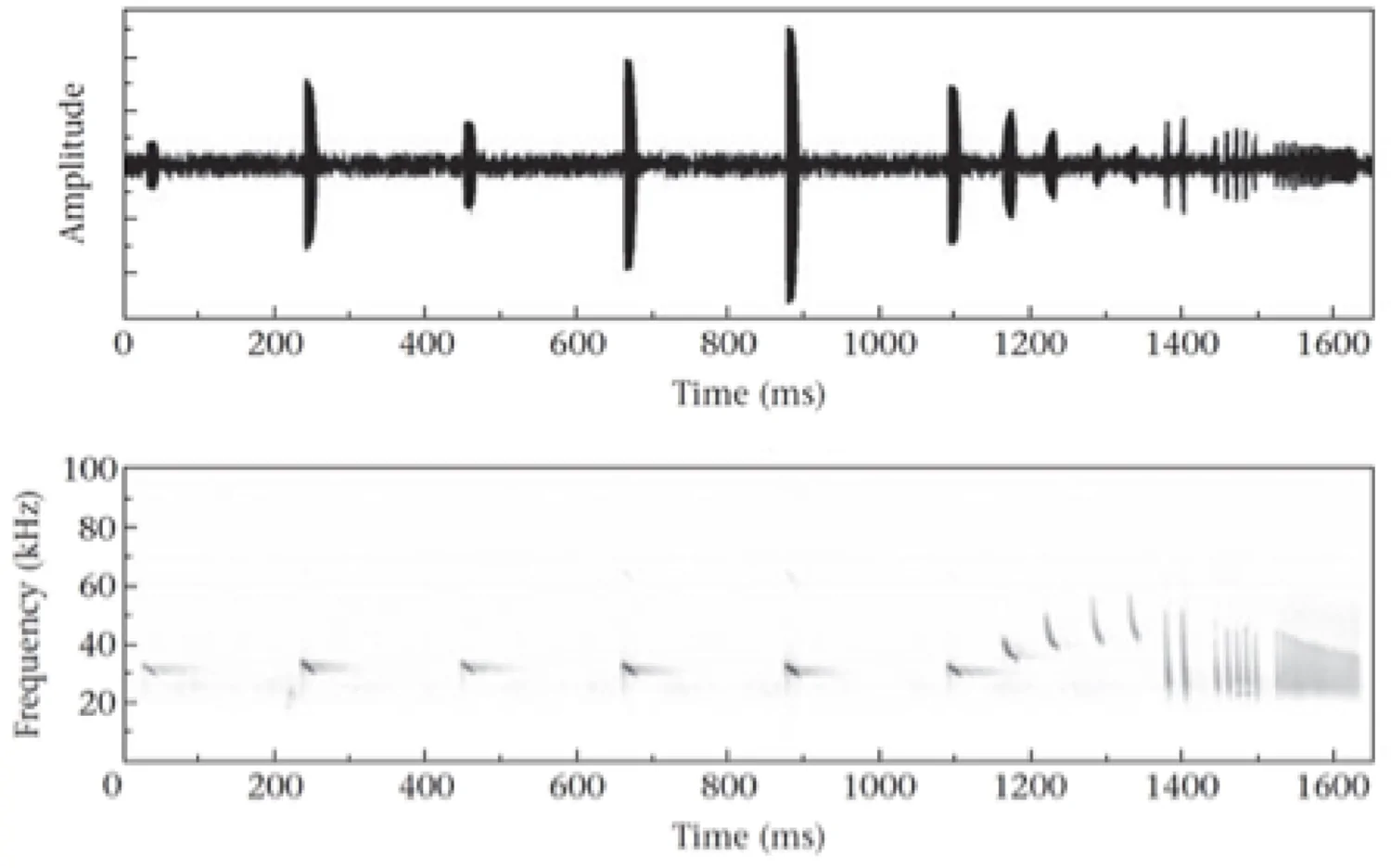
Hunting sequence of pulses emitted by a black mastiff bat (*Molossus rufus*), a high-flying insectivore, recorded by Brock Fenton in Belize. Detection apparently occurred with the echo of the shallow FM pulse emitted at 1100 ms, causing a shift to a higher frequency FM sweep, and then to much steeper FM sweeps leading into the terminal “buzz.” (Courtesy of Brock Fenton.)

A growing literature demonstrates how the emitted pulse structures of different bat species can be correlated with different habitats and foraging behavior (Schnitzler and Kalko 2001; Denzinger et al. 2016). Individual bats of many species clearly adjust their echolocation sounds to adapt to environmental conditions, acoustic clutter, and presence of other bats. What is most conspicuous, however, is the importance of behavioral context, as bats search for, pursue and catch prey. Echo detection can be enhanced by packing much of the energy in the emitted pulse into a narrow frequency band. Accordingly, the emitted “search phase” echolocation sounds of different bat species tend to be relatively narrow band and long, ranging from shallow FM sweeps lasting several ms to constant frequency (CF) or quasi-CF pulses lasting up to 100 ms or longer. After an obstacle or target is detected, however, virtually all species shorten the duration of the pulses, add or shift to a steep FM component, and increase the repetition rate. There is consensus that a wide bandwidth signal is needed to optimize distance determination, localization, and target evaluation. An elegant example of this transition is the 1.6-s-long record of the emitted sounds of a black mastiff bat (*Molossus rufus*) during search, pursuit, and (probable) capture of an insect in Belize (Fig. 1). The search pulses were shallow FM sweeps through a narrow frequency band centered just above 30 kHz, each lasting a few ms. These frequencies are probably those to which the bat is most sensitive, maximizing the likelihood of detecting the echoes of objects in the flight path and potential insect targets. After detection, the bat increased its repetition rate and shifted to progressively shorter, broader bandwidth pulses, followed by another abrupt change to still much wider bandwidth, briefer pulses leading into the terminal “buzz.” One can imagine the information content of the echoes of these distinctive emitted pulses: detection, verification of the presence of a target of interest and its approximate distance and direction, precise localization and characterization of the target, and final attack. Regardless of the interpretation of the information content of each echo, this sequence illustrates the characteristic shift from narrow bandwidth search pulses to shorter, wider bandwidth emissions at a higher repetition rate after detection as the bat located and identified the target.

Bat auditory systems are programmed to utilize downward sweeping FM sounds selectively. The big brown bat (*Eptesicus fuscus*) uses downward sweeping FM sounds for distance determination but cannot obtain distance information from upward sweeping sounds covering the same frequencies (Masters and Jacobs 1989). Broadband noise pulses are also ineffective for distance determination (Surlykke 1992), although sharply-timed tongue-generate broadband “clicks” suffice for this purpose in bats of the genus *Rousettus* (Griffin et al. 1958). Simmons (1973) established that distance discrimination is based on measurement of the elapsed time between the emitted sound and a returning echo (the “echo delay”). To a first approximation, the echo delay will be the same at all frequencies in the pulse-echo pair. Therefore, it can reasonably be postulated that as the bandwidth of a pulse increases so will the redundancy of possible measurements of echo delay, thereby increasing the accuracy of the measurement. Strong experimental evidence indicates that this is the case. In *Eptesicus fuscus* trained to respond positively to echoes at a specific delay, reduction in the bandwidth of echoes by low-pass or high-pass filtering reduced the accuracy of target detection, especially when the low frequencies in the echoes were removed (Surlykke 1992). Similarly, Bates and Simmons (2010) tested distance discrimination in *E. fuscus* using an electronic target simulator system that allowed them to manipulate the frequency content of “echoes” from phantom targets. Progressive removal of frequencies in the first harmonic by high-pass filtering caused a progressive deterioration in acuity for perception of echo delay. They concluded that the low frequencies near the end of the first harmonic sweep appeared to be disproportionately critical. Furthermore, in the presence of interfering noise, *E. fuscus* increases both the bandwidth and amplitude of its emitted pulses (Ming et al. 2021).

**Fig. 2:**
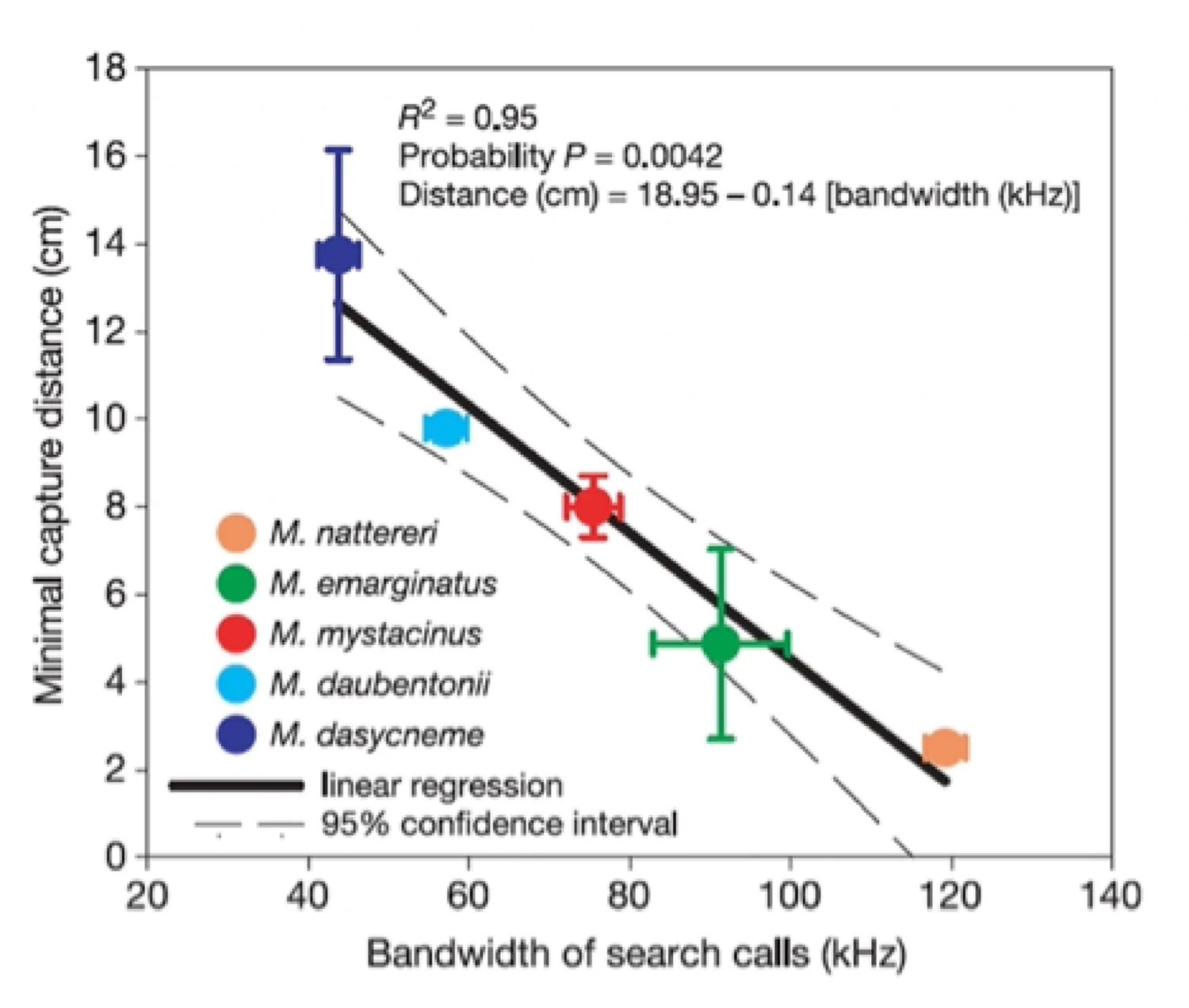
Relationship of search call bandwidth and ability to resolve and capture targets as a function of distance between the target and a background wall (from Siemers and Schnitzler 2004)

Potential differences in the bandwidth of FM sweeps and neural processing mechanisms make it challenging to compare the acuity of echolocation in different species. In a clever set of experiments, however, Siemers and Schnitzler (2004) compared five different species of European *Myotis*, all of which catch flying insects and stationary spiders near echo clutter-producing vegetation with search calls that differ in bandwidth from approximately 40 kHz (*M. desycneme*) to 120 kHz (*M. nattereri*). The minimal distance between a target and a background source of loud interfering echoes (a wall) at which the target could be captured was linearly related to the FM bandwidth employed (Fig. 2). The greater the extent of the sweep, the more accurate is the distance discrimination and ability to overcome echo “clutter” to recognize and capture prey. Capture performance was unrelated to pulse duration, peak frequency, or terminal frequency.

**Fig. 3:**
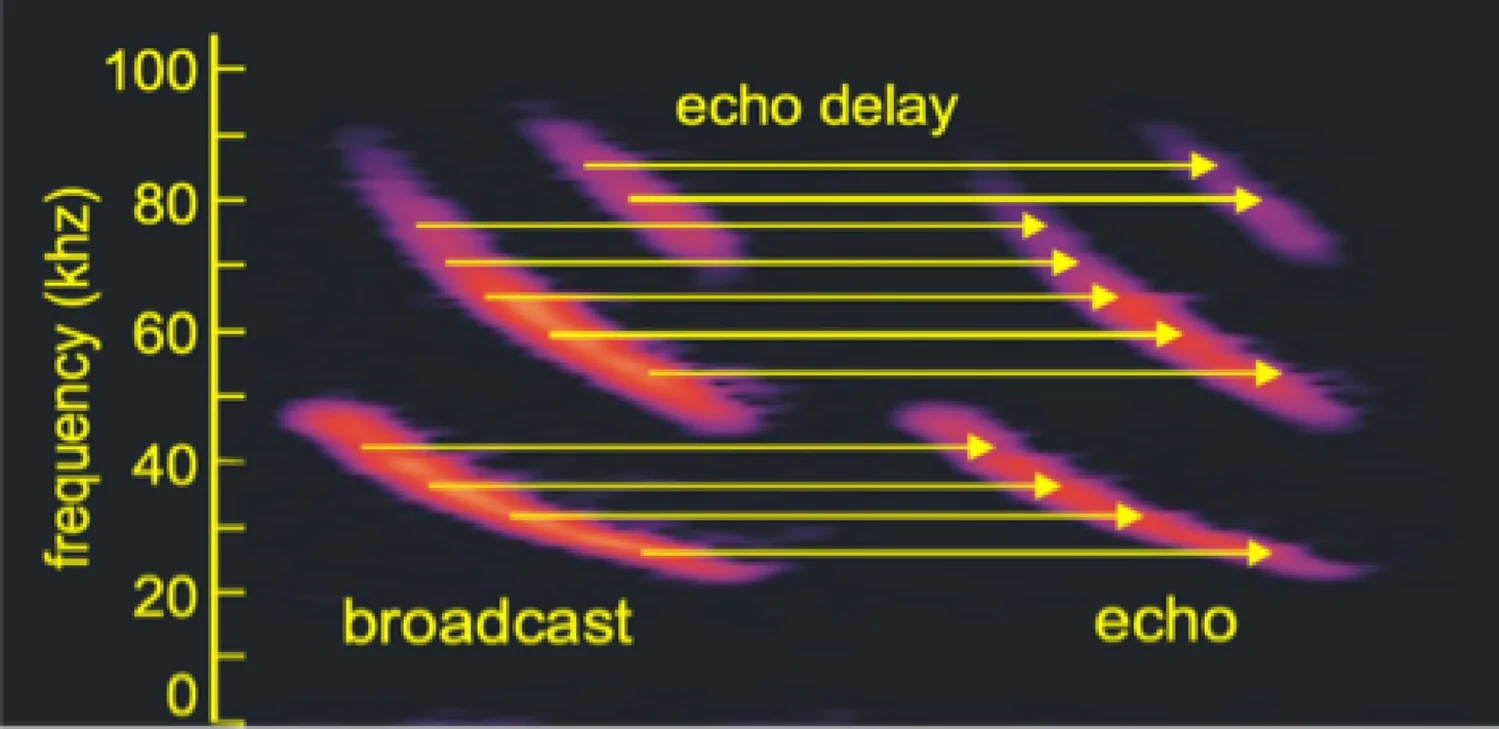
Filterbank array model of echo delay measurement at all frequencies in the returning echo (Courtesy of James Simmons)

These behavioral experiments, coupled with the almost universal shift to wider bandwidth FM sweeps after target detection, strongly support the idea that wider bandwidth sweeps increase the accuracy of distance measurement by increasing the number of overlapping bandpass filters measuring echo delay and increase the accuracy of target localization and discrimination as well. These expectations are built into a “filterbank array” or spectrogram model of distance determination (Fig. 3) (Simmons 2017). (A more detailed spectrogram correlation and transformation [SCAT] model incorporates refinements that enable extraction of multiple details about targets [Ming et al. 2021]). These models posit that each emitted pulse establishes its own template for “self-recognition” and extraction of information about targets, including target distance. Neuronal responses to sound pulses at levels above the auditory nerve are predominantly single, sharply timed action potentials that depend on the timing and intensity of the narrow band of frequencies that are excitatory for each neuron (Bodenhamer and Pollak 1981). Echolocating bats have evolved extraordinarily fast recovery of responsiveness after a loud sound and exaggerated response to a putative echo (Grinnell 1963). In the inferior colliculus, the emitted FM sweep triggers the activation of “delay-tuned” neurons that respond selectively to the same frequencies in echoes at specific delays within a range of about 2–30 ms (Feng et al. 1978; Dear et al. 1993; Dear and Suga 1995). Bats typically shorten their emitted pulses sufficiently to avoid the overlap of returning echoes with emitted pulses. Even if overlap occurs, the FM sweep typically is rapid enough that returning echoes do not overlap in instantaneous frequency (although returning echoes are likely to overlap in many environments). The models assume that temporal information is integrated over most or all frequencies, at least in the first harmonic.

Many bats emit additional harmonics that fall within their range of hearing and some, especially in the family Phyllostomidae, emit primarily higher order harmonics, with the first harmonic being almost inaudible. The amplitude and frequency structure of emitted pulses can be quite flexible–-not by altering the fundamental emitted frequency but by altering the amplitudes of different harmonics as a function of the echolocation context (Fenton et al. 2011; Vandereist et al. 2011; Luo et al. 2023; Chen et al. 2025). Lower harmonics may be emphasized for detection in open air or as clutter is decreased and higher frequencies emphasized as a way of focusing on nearby targets close to the line of flight or to increase resolution in determining target characteristics. In at least one case, the pale spear-nosed bat, *Phyllostomus discolor*, facultative biomechanical changes to the vocal tract lower the emitted frequencies slightly in response to noise, especially in higher harmonics (Luo and Wiegrebe 2016). Since the responses of many neurons in the auditory pathways are affected by more than one harmonic, changing the harmonic structure of emitted sounds (and echoes) can materially influence echolocation mechanisms (Cui et al. 2024).

Not surprisingly, the story in *Eptesicus* is even more complicated and fascinating. In their experiments modifying the echoes from phantom targets, Bates and Simmons (2010), Bates et al. (2011) found that the second harmonic contributed little to distance discrimination. By itself, the second harmonic could not support distance discrimination; in contrast, the first harmonic by itself was as effective as the combination of both. In fact, in *Eptesicus* the second harmonic seriously interfered with distance discrimination when its echo was very slightly delayed relative to those of the first harmonic. Because of frequency-dependent directionality both of sound emission and hearing, this would happen for echoes returning from objects off the line of flight. In echoes from targets in front of the bat, where the second harmonic is in near-exact temporal register with the first harmonic, the second harmonic would contribute to echo analysis; for targets significantly off the line of flight, attenuation of high frequencies would render echoes ineffective. Thus a major role of higher harmonics may be the suppression of echo interference from objects off the flight path (Bates and Simmons 2010; Bates et al. 2011). This is an important insight.

### Gating of pulse-echo delay measurements and the role of the short CF

While the filterbank array model posits narrow frequency-tuned neurons responding to the same frequencies in the emitted pulse and its echoes, measurements may be more complicated in ways that reduce the forward interference of the emitted pulses on returning echoes. For example, organized banks of cortical neurons in the long CF-FM bat *Pteronotus p. parnellii* (Parnell’s mustached bat), and possibly other long CF-FM bats, are delay-tuned to combinations of the faintly emitted fundamental (FM_1_) and echoes of the louder second, third, and even fourth FM harmonics (Suga and O’Neill 1979; O’Neill and Suga 1982).

Another interesting form of combination-dependent response emerged from experiments with the lesser bulldog bat, *Noctilio albiventris* (Roverud and Grinnell 1985a, 1985b). This species emits short CF-FM search pulses, typically in pairs, at an onset interval of about 28 ms. The first is a quasi-CF pulse of 72–75 kHz and ~ 8 ms duration; the second consists of a ~ 6–8 ms-long CF component of 72–75 kHz that terminates in a 2–4 ms downward FM sweep to about 50 kHz (Kalko et al. 1998). The FM component of the second pulse in the pair is presumably critical for target distance determination. The function of the CF component is an issue that I wish to consider further.

**Fig. 4:**
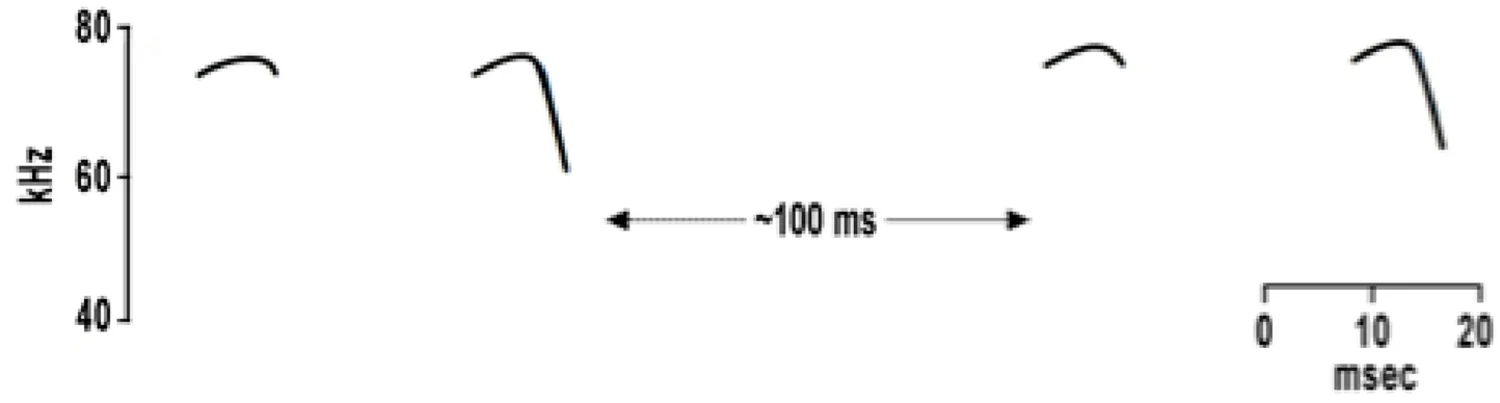
Sonograms of characteristic emitted pulses of *N. albiventris* in the laboratory while making distance discrimination decisions. Quasi CF and CF-FM pulses separated by ~ 27 ms were emitted in pairs at a repetition rate of approximately 10/s. (Adapted from Roverud and Grinnell 1985a)

In these experiments, two *N. albiventris* were trained to discriminate the closer of two targets at a distance of 35 and 40 cm. They typically emitted pairs of CF/CF-FM sounds at a rate of 7–10/s and quickly learned to make the distance discrimination at a success rate of more than 90%. Typical emitted pulses in the laboratory distance discrimination experiments are shown in Fig. 4.

The initial aim was to determine whether artificially generated pulses that simulated the bats’ own emitted pulses (10 ms 75 kHz CF ending in a 2-ms, 25 kHz downward sweep to 50 kHz) could be used by the bats for distance discrimination. This hypothesis proved difficult to test because the bats insisted on emitting their own pulses. However, the artificial pulses, free-running at a repetition rate of 10/s and an intensity of 91 dB (approximately 20 dB fainter than the bats’ own emissions and about 30 dB louder than echoes from either target), blocked the bats’ ability to discriminate distance using echoes of their own sounds. This made it possible to analyze the features of the artificial sounds that were critical to this interference. Masking could not explain the phenomenon because the artificial pulses rarely overlapped in time with the natural pulse-echo pair; nor did white noise pulses of the same duration, intensity, and repetition rate interfere with distance discrimination. Interestingly, altering the repetition rate of the artificially simulated bat calls suggested that at least two successive natural information-bearing CF-FM pulse-echo pairs without an interfering pulse were necessary for distance discrimination. Presenting the bats with silent windows of different duration in otherwise solid masking white noise led to a similar conclusion (Roverud and Grinnell 1985a). CF-FM pulses simulating the bats’ own sounds did not interfere with detection or with left–right localization of a target, both of which can apparently be achieved with a single pulse-echo pair.

**Fig. 5:**
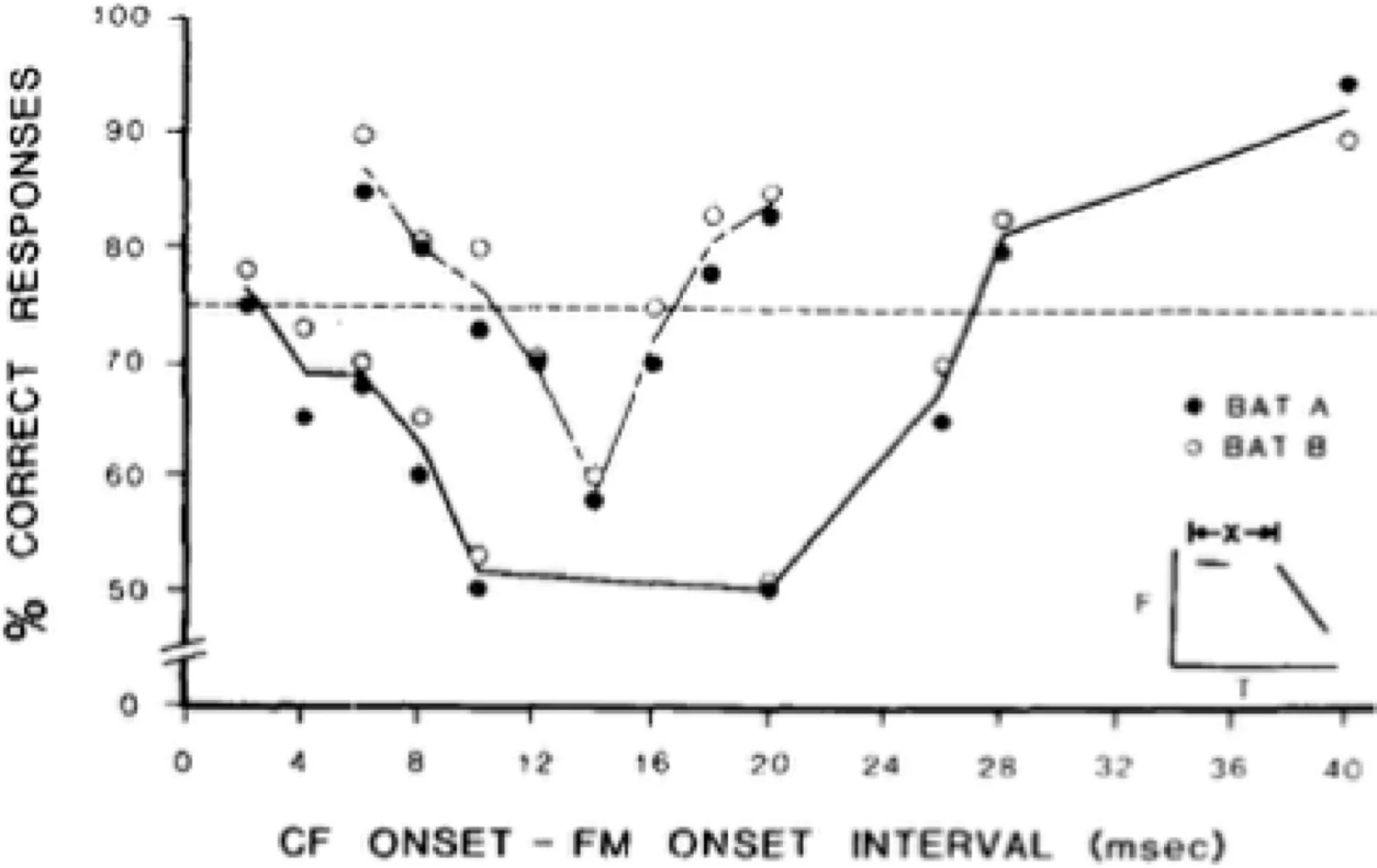
Interfering effectiveness of 10/s free-running CF-FM artificial echoes as a function of timing of a 2-ms CF component at 75 kHz preceding a 75–50 kHz FM sweep. Lower curve: interfering signal at 91 dB intensity at the bats’ listening position; upper curve, simulated echo intensity reduced to 81 dB SPL. Data from two bats. 75% correct was arbitrarily selected as the criterion value for discrimination (from Roverud and Grinnell 1985b)

Significantly, neither free-running CF nor FM components alone interfered with distance discrimination. For interference to occur, the artificial pulses had to have both CF and FM components and the CF component had to be between about 2 and 27 ms in duration. However, the CF component could be reduced to as little as 1 ms in duration if it was presented 8–25 ms before the beginning of the FM sweep. A 1-ms CF component continuous with the FM sweep failed to interfere. When the intensity of the artificially simulated echo was reduced from 91 to 81 dB SPL, the optimal time for a brief CF component to occur was between 10 and 16 ms before the beginning of the FM sweep (Fig. 5). The CF component appears to trigger a temporal gate of 25–30 ms duration during which an FM sweep is interpreted as carrying distance information that can interfere with normal echolocation. Thirty ms corresponds to a target distance of approximately 5 m, the likely working distance of echolocation in this and many other species of bats. Echoes or other sounds arriving outside this time window would be treated as clutter.

The interfering effectiveness of the CF-FM artificial echoes was surprisingly tolerant of changes in the absolute frequencies presented to the bats. If the bandwidth of the artificial echo FM sweep was reduced by truncating the low frequencies, the signal continued to interfere with distance discrimination until it was reduced to about 11 kHz, from 75–64 kHz. Moreover, the simulated CF-FM echoes were equally effective at interfering even if they were shifted downward in frequency from the normal 75 kHz CF to as low as a CF of 55 kHz ending in a 25 kHz sweep to 30 kHz, mostly below the normal emitted frequencies. In contrast, shifting the whole signal upward from a CF of 75 kHz to 76 kHz caused an immediate sharp drop in effectiveness, and interference disappeared altogether if the CF was as high as 80 kHz, even though the ensuing FM component swept through most of the normal FM range. To cause interference effectively, the FM sweep had to begin at the same frequency as the CF component. Interference was significantly reduced when the FM sweep began as little as 1 kHz above or 3 kHz below the normal 75 kHz CF. The emitted CF clearly serves as a gate-opening signal because artificial FM sweeps alone, which are ineffective when free running, do interfere with distance discrimination when they are coupled to the emitted pulse and occur within the ~ 27-ms window opened by the emitted pulse CF (Roverud and Grinnell 1985b).

This time window corresponds to the time when the emitted FM sweep occurs and returning FM echoes are processed by the auditory nervous system and populations of delay-tuned neurons are programmed to make delay measurements. Presumably, when a sufficiently loud artificial CF-FM “echo” arrives, uncoupled to the bat’s own emission, it activates the same circuitry in the bat’s brain. A time window containing an initial FM sweep is opened and the banks of frequency and delay tuned neurons are somehow programmed, or “sensitized”, to make accurate measurement of echo delay at each frequency in the sweep, but no meaningful echoes follow. An FM sweep without the preceding CF trigger or outside of the time window established by the CF component does not activate the circuitry to make those time measurements. As Fig. 5 shows, when the CF component precedes the FM sweep by as much as 25 ms, the window is already closing and interference is considerably reduced.

What then is the nature of this programming or “sensitization” of the banks of frequency and delay-tuned filters? Delay-tuned neurons in the inferior colliculus and thalamus of the mustache bat *Pteronotus parnellii* are, at least in part, the result of GABAergic inhibition that can last as long as 30 ms, triggered by response to the emitted sound (Park and Pollak 1993; Saitoh and Suga 1995; Yan and Suga 1996; Butman and Suga 2019. At least in sub-cortical pathways, glycinergic inhibition appears to play a major role [Wenstrup et al. 2012]). When the short latency response to a given frequency in the echo coincides with the long latency recovery from inhibition triggered by the same frequency in the emitted sound, response of those delay-tuned neurons is strongly facilitated. The delay tuning of neurons for 25–30 ms in the inferior colliculus of *E. fuscus* after pulse emission probably arises in the same way (Ferragamo et al. 1998). Delay tuning in a large fraction of *E. fuscus* inferior colliculus neurons also occurs with extrinsic presentation of pulse-echo pairs, as long as they occur in a quasi-natural echolocation sequence or at a sufficiently rapid repetition rate, but not to trains of FM pulses or echoes only, single pulse-echo pairs, or trains of pairs separated by as much as 250 ms (Macías et al. 2019). This suggests a dependence on some kind of central gating for delay tuning, perhaps in descending inputs from the auditory cortex in this FM bat as well. By analogy with these findings, the loud CF component of the normal emitted pulse in *N. albiventris* appears to be necessary to trigger echo delay measurement, probably at least in part by enabling the outgoing FM to initiate inhibition of variable duration, from 2 ms up to 25–30 ms, in each bandpass-tuned population. Recovery from inhibition, coinciding with excitation by the same frequencies in the echo FM sweep, then facilitates responses at different delays characteristic of the delay-tuned neurons. The time course of interference in Fig. 5 apparently reflects the time course of effectiveness of the CF in enabling the FM component to initiate the delay measurement process.

### Number and properties of the narrow bandwidth “filters” that extract distance information from the FM sweep

Returning to the filterbank model for extraction of distance information from the FM sweep, major questions remain. What is meant by “redundant measurements?” How many bandpass “filters” are there and what are their properties? (I am purposely avoiding the issue of how precise the measurement is–whether it requires phase-resolving capability and µs or better-distance resolution.) Based on the sharpness of tuning curves and difference limens in *E. fuscus,* von Stebut and Schmidt (2001) estimated that the bandwidth of individual frequency channels in that species is 1–2% of the center frequency of the sweep, or 50–100 channels. What is the nature of the circuity that integrates temporal information from a wide bandwidth of frequencies? What is the minimum frequency bandwidth necessary for a bat to obtain distance information?

Innovative experiments done by Roverud with the lesser bulldog bat, *N. albiventris,* and with the big brown bat, *E. fuscus*, provide insights relevant to these questions. His findings suggest that while emitted FM sweeps may differ greatly in bandwidth and duration, a minimum number of discrete frequency filters or channels is required, largely independent of the absolute frequencies or the frequency separation between frequency-tuned filters. These experiments also provide valuable information about other properties of the frequency- and delay-tuned filters (Roverud 1993, 1994).

When hanging on a perch, *N. albiventris* regularly emit a series of pulse pairs (quasi-CF and CF-FM) at a repetition rate of about 5–10/s (as they appear to survey the environment acoustically. When an object is moved toward them, they usually react by dropping the quasi-CF emitted pulses and increasing the repetition rate of the CF-FM pulses to 30/s or higher–-behavior similar to when they detect a target in flight.

Roverud (1993) showed that artificial “echoes” produced electronically and manipulated by the experimenter could evoke the same behavior. Bats were trained to hang from a perch at one end of a dimly lit flight room. A nearby microphone picked up the bat’s vocalizations. When several pulses had been emitted at the normal search rate, the next emitted pulse triggered an electronically generated virtual echo from a loudspeaker at a delay of 30 ms, corresponding to a target distance of 5 m, and each successive emission by the bat triggered another virtual echo at a progressively shorter delay simulating a target approaching at 5 m/s and disappearing after reaching a distance of about 0.3 m. If the bat’s emissions followed the normal approach behavior of increasing to an emission rate of about 30/s for at least six pulses, the response was judged positive. That is, the bat had interpreted the virtual echo as a real echo from an approaching target. If the bat did not display an approach phase response, the trail was judged negative. Aside from training the bat to hang from the perch, no further training and no rewards were involved. When artificially generated CF-FM “echoes” simulating the normal CF-FM component of search pulses of *N. albiventris* were played back through the loudspeaker at the diminishing intervals, approach phase responses were elicited about 70% of the time**.** Why the success rate was not 100% is a valid question, but it should not be considered surprising. Approach phase responses depend on the bat’s mood and on whether or not it is interested in tracking targets. Regardless, the response rate of about 70% was consistently found to be the maximum percentage of positive approach phase responses in control experiments with artificial echoes simulating the bats own CF-FM signals.

**Fig. 6:**
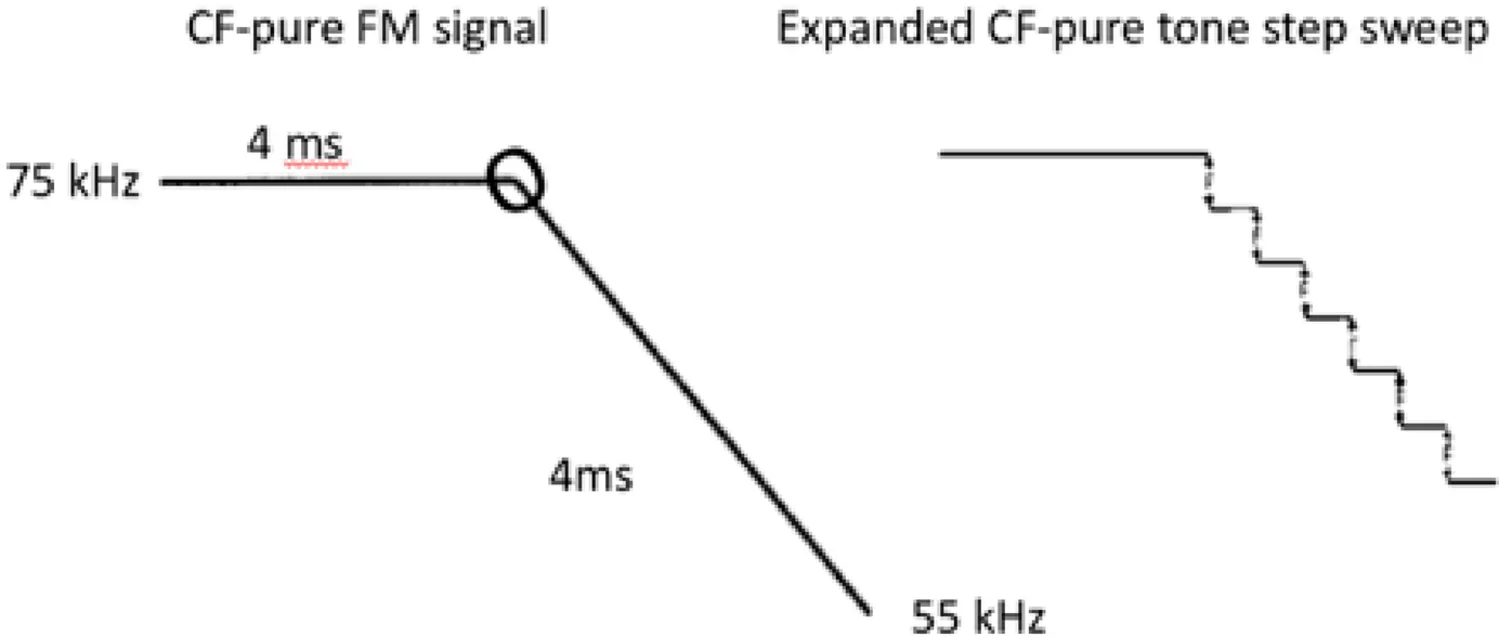
Diagrammatic representation of pure-tone artificial echoes used in experiments. The smooth FM sweep was replaced with a series of brief pure tone steps that could be altered in number, step size, and duration

The initial aim was to determine how many frequencies in the FM sweep were necessary to elicit a positive response. In a novel approach, electronically generated artificial echoes were produced consisting of 4 ms CF at 75 kHz, similar to typical emitted pulses, but instead of being followed by the normal smooth 2–4 ms downward sweep to 55 kHz, the “FM” component consisted of a series of discrete pure tone frequency steps, digitally switched from step to step seamlessly, descending to 55 kHz in 4 ms (Fig. 6). Seamless switching was done in < 500 ns, maintaining the phase and amplitude of the end of one pure tone step and the beginning of the next. Frequency spectra of the pure tone steps showed that the energy in the signal was largely confined to the frequencies of the pure tone steps. All experiments were done with 5 bats.

**Fig. 7:**
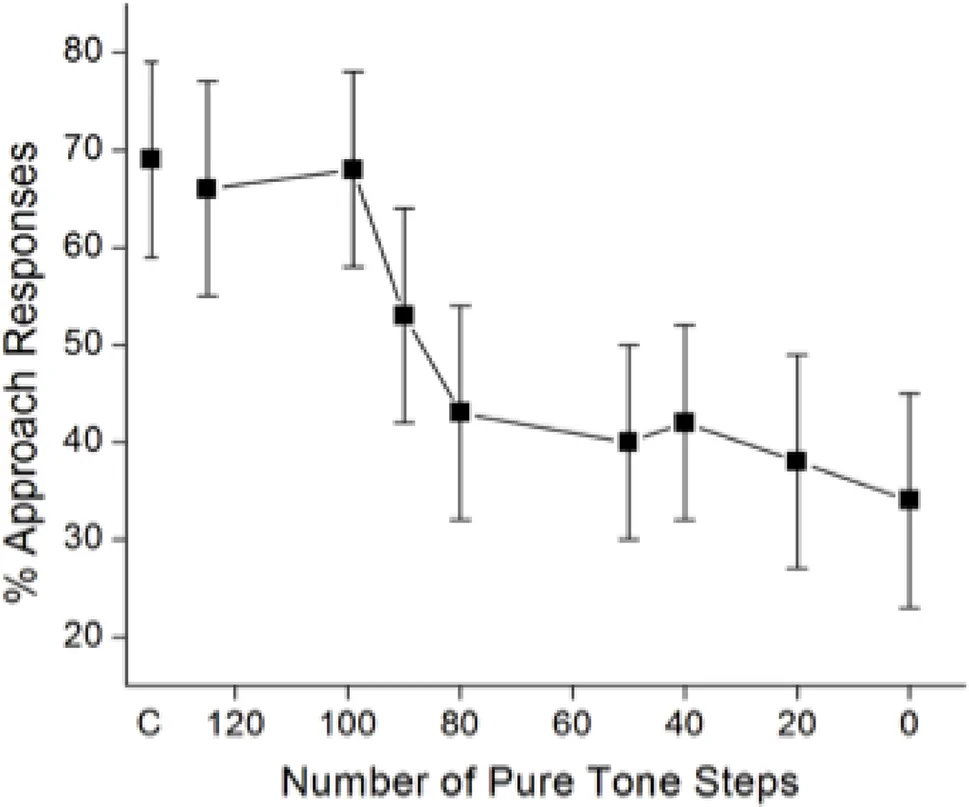
Effectiveness in eliciting an approach phase response of virtual echoes in which the 4 ms smooth FM sweep from 75 to 55 kHz (control, C) was replaced by virtual “FM sweeps” consisting of the number with pure tone steps shown. In all cases, the “FM” was preceded by a 4-ms CF component at 75 kHz. Because the FM component was always 4 ms in duration, the step durations varied from 32 µsec (125 steps) to 200 µsec (20 steps). Because the FM component swept from 75 to 55 kHz in all cases, the step size varied from 160 Hz (125 steps) to 1 kHz (20 steps). The zero step point represents an artificial “echo” with no FM “sweep”, only 8 ms of CF. Plotted are the means ± SD of data from 5 bats, each tested 20 times at each step number. (Adapted from Roverud 1993)

When the continuous 4 ms sweep from 75 to 55 kHz (C) was replaced by 99 or more pure tone steps, the artificial “echoes” were as effective in eliciting approach phase responses as a CF-FM “echo” with a smooth FM sweep, while 90 steps were less effective, and 80 or fewer steps were almost as ineffective as a 75 kHz CF component that did not step down at all, eliciting approach phase emission behavior about 40% of the time (Fig. 7). These results suggest that a minimum number of 90–100 discrete frequency channels must be activated by an echo for it to be interpreted as being an echo of the bats’ own emissions and tracked for target distance. However, in this experiment, when the number of steps was varied from 125 to 20 steps in 4 ms, the durations of the steps changed from 32 µs to 200 µs per step and their sizes varied from 160 Hz to 1000 Hz per step. Was it truly the number of steps or the size or duration of each step and their effect on the bandwidth and rate of FM sweep that made the difference?

To test the importance of the overall frequency range of the FM sweep, the *N. albiventris* were tested with artificial echoes consisting of the usual 4-ms, 75-kHz CF component followed by 4-ms sweeps composed of either 99 or 80 pure tone steps of 100, 200, and 400 Hz to simulate an FM sweep through 10, 20 and 40 kHz (99 steps) and 8, 16, and 32 kHz (80 steps), respectively. The 99-step sweeps elicited approach phase behavior at all step sizes more than 65% of the time–-as effectively as the control smooth FM sweep from 75–55 kHz. In contrast, the 80-step pure tone sweeps of all three sizes were ineffective at eliciting positive responses. On the other hand, sweeps of 99 steps of 50 Hz each (~ 5 kHz total sweep) were considerably less effective than 100 Hz, eliciting responses only 49% of the time. Apparently, the step size needs to be larger than 50 Hz to be usable as one of the 90–100 steps. However, a 5-kHz sweep from 75–70 kHz (100 steps of 50 Hz each) may be insufficient bandwidth to simulate a true 20-kHz FM sweep. To resolve this question, a similar experiment was performed with 99 pure tone steps in which every odd-numbered step (1,3,5, etc.) was either 50, 100, 200, 300, or 350 Hz, while even-numbered steps were 350, 300, 200, 100, and 50 Hz. In all cases the average step length was 200 Hz, and the total sweep length was from 75 to 55 kHz. Each step was 40 µs in duration. The sweeps including 50-Hz steps evoked few responses above chance; the sweeps composed of only steps of 100–300 Hz solicited responses a mean of 60–67% of the time, clearly leading the bats to react as if the “echoes” were carrying valid distance information (Roverud 1993). Thus an FM sweep in *N. albiventris*, to be interpreted as a true echo, must contain 90 or more pure tone steps, individual steps must be > 50 Hz, and the individual frequency-specific channels can be distributed over a bandwidth as small as 10 kHz (75–65 kHz) to be viewed as carrying distance information.

To explore the importance of the duration of individual steps or of the total sweep, Roverud (1993) presented the five *N. albiventris* with 75–55 kHz sweeps consisting of 99 or 80 pure tone steps with individual step durations of 20, 40, and 80 µs. Total sweep durations were 2, 4, and 8 ms for the 99-step sweeps and 1.6, 3.2, and 6.4 ms for the 80-step sweeps. At all step durations, the 99-step sweeps elicited positive approach phase responses at a rate of 65–70% compared to ~ 40% of the time when the bats were exposed to any of the 80-step sweeps. A 40-µs segment at 75-kHz corresponds to three cycles. At 65 kHz the segment is 2.6 cycles, and it is 2.2 cycles at 55 kHz. A 20-µs segment of a 55-kHz sound is only 1.1 cycles long. Apparently, 1–2 cycles are sufficient for the bats to discern a distinct frequency.

**Fig. 8:**
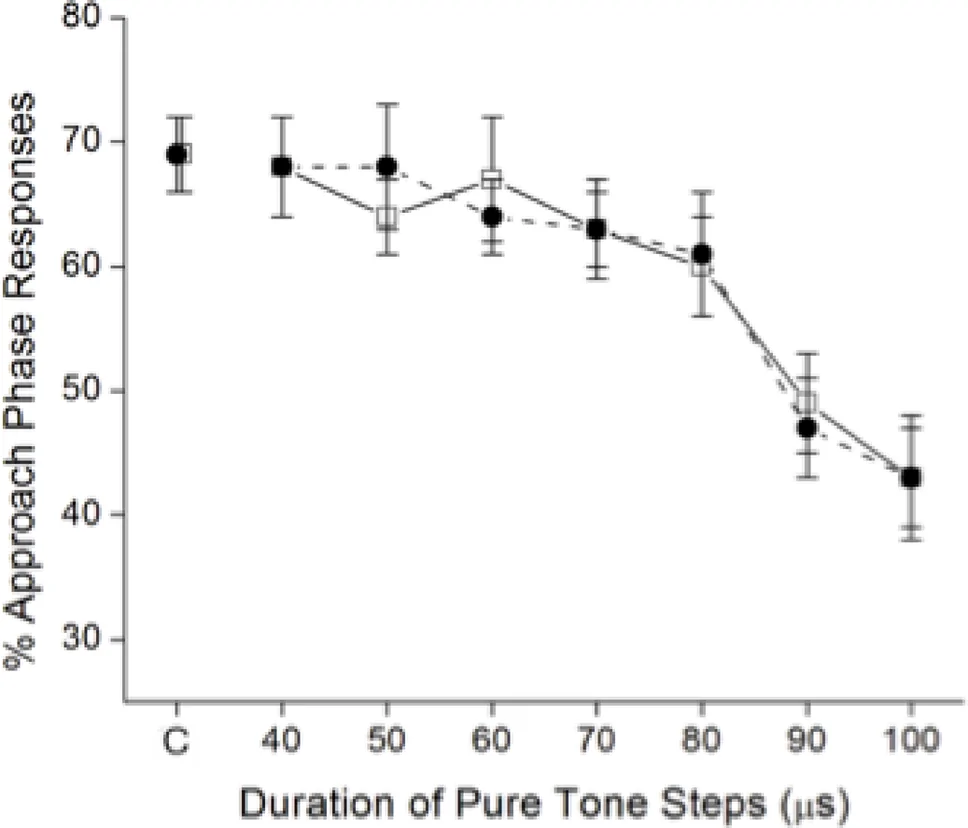
To be effective in eliciting an approach phase response in *N. albiventris*, individual pure tone steps must be 80 µs of shorter in duration. The dashed line shows the effects of increasing the duration of individual pure tone frequency steps (98 steps, each 200 Hz in size) in eliciting an approach phase response (mean ± SEM for 5 bats). The solid line shows that there was no significant difference when the duration of each odd-numbered step was always 40 µs but the even-numbered steps were of the duration shown. Similar results were found at step sizes of 100 and 400 Hz (adapted from Roverud 1994)

On the other hand, there was an upper limit to the duration of pure tone steps sweeping from 75 to 55 kHz that would evoke a stereotyped approach phase response. When 98 pure steps, either 100, 200, or 400 Hz in size, were increased beyond 80 µs to 90 and 100 µs duration, their efficacy in eliciting an approach phase response decreased sharply (Fig. 8, dashed line). To test whether this was really a limit to the duration of each step or to the total duration of the pure tone sweep (~ 4 ms for a step duration of 40 µs, ~ 8 ms for an 80 µs duration, ~ 9 ms for 90 µs steps), similar experiments were done with every odd-numbered step being 40 µs in duration, while even numbered steps varied (Fig. 8, solid line). Again, independent of the overall duration of the sweep, when the even numbered sweeps were up to 80 µs in duration, the bats’ performance was not significantly different than with 40 µs steps or with a continuous FM sweep. However, when every other step was 90 or 100 µs, the percentage of trials eliciting an approach phase response was severely reduced (Roverud 1994). Apparently individual frequency steps can be no longer than about 80 µs to still be recognized as one of the minimum of 95–100 individual frequency channels contributing to echo delay measurement. In a given frequency filter, 80 µs may be the limit of integration time for registration of echo delay.

### Properties of frequency and delay tuned filters in *Eptesicus fuscus*

Similar experiments performed with three big brown bats (*E. fuscus*), a purely “FM bat” species, showed interesting differences (Roverud and Rabitoy 1995). While hanging from a perch in the laboratory setting, these bats intermittently scanned their environment by emitting a burst of FM pulses at a repetition rate of ~ 5/sec, each approximately 4 ms long. These pulses contained a first harmonic that swept from about 55 to 25 kHz and a second harmonic that contained significant energy between ~ 85 and 55 kHz. When an *E. fuscus* detects an approaching target, it increases its pulse emission rate to ~ 13/sec. This approach phase response was used as a criterion for whether the bat had interpreted an artificial echo as being a real echo reflecting the existence of a target moving toward it. As in *N. albiventris*, this behavior was not elicited by every test. It occurred about 60–65% of the time even when the returning “echoes” were smooth FM sweeps from 75 to 25 kHz in 4 ms that simulated their own emitted calls. Nevertheless, with this as a control value, the effectiveness FM sweeps composed of pure tone steps could be tested.

**Fig. 9:**
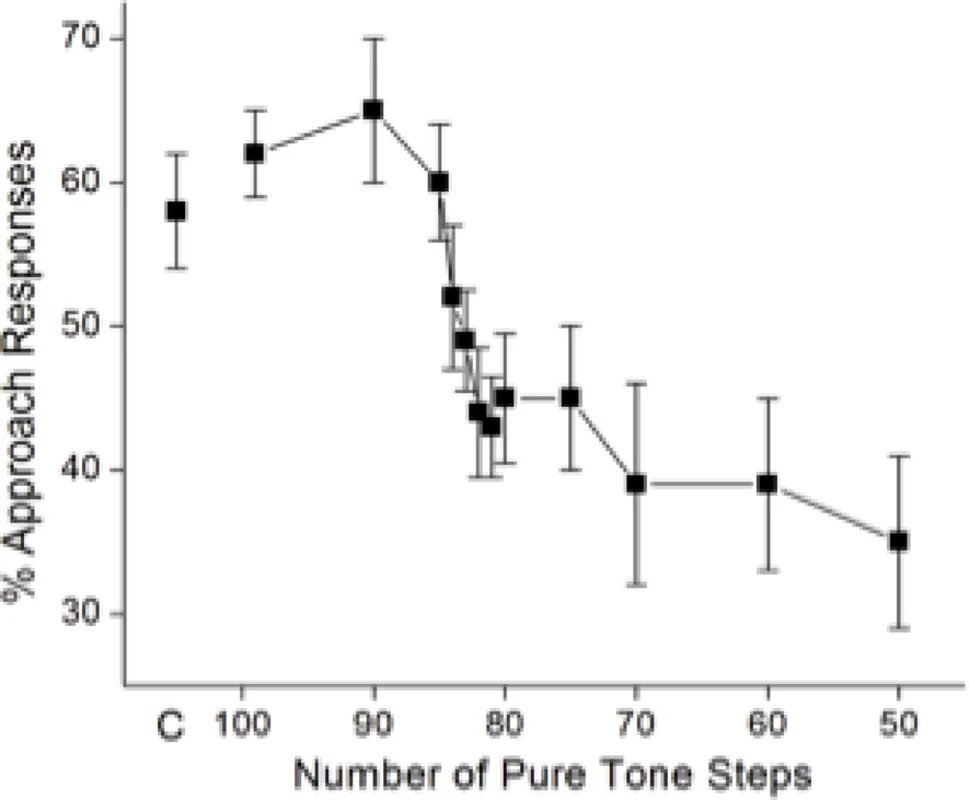
Effectiveness of artificial echoes consisting of different numbers of pure tone steps evenly spaced between 75 and 25 kHz in evoking an approach phase response in three *E. fuscus* bats (mean ± SEM). The individual pure tone steps were varied in size and duration to maintain a 4-ms duration sweep. C, the percentage of response phase responses to the artificial control echoes consisting of a 4 ms continuous FM sweep from 75 to 25 kHz. (Data from Roverud and Rabitoy, 1994)

In these experiments, as in those with *N. albiventris*, when an *E. fuscus* hanging from a perch emitted pulses at a resting rate, artificial echoes coupled to those emissions were presented at decreasing delays to simulate a target that first appeared at a distance of 5 m and then approached the bat at a rate of 5 m/s. Each artificial echo consisted either of an actual 4 ms-long, continuous FM sweep from 75–25 kHz (the control) or a series of pure tone steps, each seamlessly merged in phase and amplitude with the next, in a downward sweep from 75–25 kHz. The duration of the sweep was always 4 ms, but the number of pure tone steps varied from ~ 100 to 50. There was a sharp decrease in effectiveness at evoking an approach phase response when the number of frequency steps was decreased from 85 to 82 (Fig. 9).

In this experiment, the total “echo” duration was held at 4 ms and the sweep in all cases was from 75–25 kHz, so as the number of pure tone steps was varied the step size and duration changed systematically from about 505 Hz/step, each ~ 40 µs in duration (99 steps), to 1000 Hz/step, each 80 µs in duration (50 steps). To determine whether the drop in effectiveness with fewer steps was the result of the number, size, or duration of the steps, additional experiments were performed with the same types of controls as in the experiments with *N. albiventris*. The results showed that 83 or more pure tone frequency steps were required to elicit a positive approach phase response, and the steps had to be 450 Hz or greater in size. Step durations of 40 or 50 µs were effective in eliciting a positive response, but 60 µs or longer steps were not. These values are compared with those obtained for *N. albiventris* in Table 1.

**Table 1:** Properties of pure tone steps in FM “sweeps” necessary to evoke an approach phase response when presented to bats as artificial echoes of emitted pulses at delays simulating a target approaching at 5 m/s

Species	Minimum number	Minimum step size	Maximum duration
*Noctilio albiventris*	~ 95	100 Hz	80 µs
*Eptesicus fuscus*	~ 83	450 Hz	50 µs

The FM sweeps in *N. albiventris* were preceded by 4 ms CF at 75 kHz; in *E. fuscus*, the 4 ms FM sweep was linear and always started at 75 kHz

## Discussion

The experiments on interference with distance discrimination in *N. albiventris* show that for echoes to be interpreted as carrying distance information in this short CF-FM bat*,* the FM sweep must be preceded by a CF component at the same frequency as the beginning of the FM sweep. Furthermore, the CF component must occur a minimum of 2 ms before the FM sweep, is maximally effective 10–15 ms before, and is still effective more than 20 ms before the sweep. The CF component somehow enables the nervous system to quantify the time between the occurrence of each frequency in the emitted FM and return of the same frequency in echoes, as long as they return within 25–30 ms. The trigger cannot be an efferent signal coincident with the emission of the CF component, since the artificial CF-FM sounds also trigger it. The CF component itself seems to alert the auditory nervous system to the imminent FM sweep and ready it to make delay measurements starting at the frequency of the CF. How it does this is obviously a subject for further research. Possibly it causes the auditory cortex to feed back a command to the midbrain opening the window for time measurement. The required minimum 2 ms or greater interval between the onset of the and the beginning of the FM provides time for at least one additional synapse in the circuit. Early findings in another short CF-FM bat, *Pteronotus davyi*, which exhibits phenomenally rapid, robust recovery of responsiveness (Grinnell 1970), provides evidence suggestive of cortical feedback which could be related to distance determination. Microelectrode recordings of local populations of inferior colliculus neurons showed late (up to 18 ms latency), sharply tuned bursts of activity to pure tone pulses at the CF of the bat’s sounds, at intensities up to 30 dB fainter than the much more broadly tuned excitatory response thresholds of the same neurons (Grinnell and Brown 1978). These unexplained bursts might represent powerful recovery from inhibition even in the absence of echoes.

The measurement of delay presumably is initiated by the time of occurrence of each frequency in the emitted FM sweep. It also seems likely that the emitted FM sweep initiates the inhibition of populations of delay-tuned neurons which recover from inhibition after different lengths of time to evoke facilitated responses when the same frequencies in echoes coincide with their recovery from inhibition. However, without the CF component, this period of inhibition and variable timing of recovery from inhibition apparently do not occur. The CF component, whether emitted or heard in an artificial CF-FM sound, is necessary.

The findings that the FM sweep must start at the same frequency as the CF, and that interference persisted even when both the CF and FM components of the free-running artificial “echoes” were shifted downward in frequency by as much as 20 kHz, as long as they were shifted together, suggest that the CF somehow selects and aligns the banks of filters to be used in making the delay measurement? (That the CF-FM signals cannot be shifted upward in frequency more than 2–3 kHz without losing effectiveness is not surprising because that would probably move the CF component out of the bat’s response area, and the FM sweep, by itself, does not interfere.) The question remains: what is the neural circuitry that requires the combination of the emitted CF and following FM sweep to program the neuronal populations in the midbrain to measure echo delays? Is it a more obvious equivalent of the postulated central gate in the FM bat *Eptesicus fuscus* that governs the effectiveness of the FM sweep in activating delay-tined neurons, depending on the pattern and pulse repetition rate of pulse-echo pairs (Macías et al. 2019)?

This discussion has not addressed the question of what the first, quasi-CF pulse of the pair regularly emitted by N. albiventris in search flight does for their echolocation. The only certainty is that it has a function. It is probably significant that the onset interval between the two pulses in the pair is about 28 ms—the duration of the gated widow opened by the CF. One can speculate, for example, that the first of the pair not only aids in detection of targets and but also entrains the activity state of delay measuring neurons to ensure that they are optimally prepared to measure distance with the following CF-FM signal. But there is no experimental evidence that bears on the question. The first of the pair is dropped during the approach phase, and the interfering CF-FM artificial pulses simulated only the second of the pair.

These results also raise the important question of whether the CF component in long CF-FM bats might play a comparable role. In these bats, multiple functions of the long CF component are already clear. The auditory nervous system is extremely sharply tuned around the frequency of the CF component, allowing them to obtain vital information about target wing-beat characteristics, closing speed, and more. Roverud (1989), working in Neuweiler’s laboratory, repeated the kinds of experiments he had done with *N. albiventris* on one of these long CF-FM species, *Rhinolophus rouxi*, the rufous horseshoe bat. This species emits multi-harmonic pulses with a dominant second harmonic CF component of ~ 75 kHz lasting about 40 ms, preceded by a brief upward FM component and followed by a ~ 2 ms-long downward FM sweep to about 65 kHz. Two *R. rouxi* were trained to make an easy distance discrimination and Roverud tested the effectiveness of 10/s free-running artificial pulses simulating their own in interfering with this distance discrimination. The results were similar to those with *N. albiventris*, but with a much longer time window of interference. Loud free-running 10/s artificial CF pulses (45 ms duration, 75 kHz) by themselves did not interfere with distance discrimination, nor did FM signals simulating those in the bats’ own emissions without the preceding CF. Artificial signals combining the CF and downward FM sweep, however, were highly effective. As in *N. albiventris*, the FM needed to start at the frequency of the CF, and the CF could be as brief as only about 2 ms in duration as long as it preceded the FM sweep by anywhere from 2 to 60 ms. FM sweeps without the preceding CF were effective in interfering if they were coupled with the bats own emissions and timed to come within the 25 ms after the bat’s emissions, but not during the emitted CF. In short, the CF plays a gating function for distance discrimination in this *Rhinolophus* species as well.

Then the question is whether the same might be true of all CF-emitting bats, including *Pteronotus parnellii* which, as mentioned above, has been studied extensively by Suga and his colleagues. The remarkable organization of the auditory cortex that they have described, with parallel maps of target distance based on combination-sensitive neurons measuring the delay between frequencies in the (mostly inaudible) emitted FM_1_ and the same frequencies in the echo FM_2_ FM_3,_ and FM_4_, were based on presentation of pairs of multi-harmonic FM or CF stimuli, simulating those components in the emitted pulses and echoes, but not combinations of the two (CF-FM pairs), which would more closely simulate the actual pulse-echo pairs (O’Neill and Suga1982; Suga 2015). The possible role of the CF component of the emitted calls in gating a time window for distance discrimination has not been tested in *P. parnellii*. It seems quite possible, however–even probable–that the long CF in *P. parnellii* also plays that role. It would be extremely interesting to examine the effects of coupling the preceding CF component to the FM sweeps on response patterns of neurons in the auditory pathways and on auditory pathway organization in *P. parnellii*, as well as any other CF-FM bats. Also, research is needed in *N. albiventris*,or in other short CF-FM bats with more prominent harmonics, to determine whether there is evidence for distance discrimination involving responses to combinations of harmonics, as in *P. parnellii*.

Interpretation of the experiments on the effectiveness of a descending series of pure-tone steps in evoking normal approach phase responses is subject to the caveats that the artificial echoes were not actual copies of the emitted sounds and that the responses clearly depended on the bat’s level of interest at the time of testing, given that both species only responded to actual continuous FM sweeps simulating their own pulses in ~ 60–70% of the trials. Nevertheless, multiple bats of both species yielded closely similar, statistically significant data that reveal several interesting properties of the bats’ narrowly frequency-tuned, delay-measuring channels.

Most novel was the identification of an absolute minimum number of pure tone steps needed for the simulation to work, that is, to deceive the bat into reacting as it would to a real approaching object–-95–100 in *N. albiventris* and 82–83 in *E. fuscus*. The effectiveness did not degrade gradually with reduction in the number of steps but failed relatively abruptly. The size and duration of the steps could vary considerably without changing the minimum required number of steps. The most dramatic difference between the two species was in the minimum step size (100 Hz in *N. albiventris* and 450 Hz in *E. fuscus*). which presumably corresponds to the frequency-resolving ability of the auditory nervous system and reflects the sharpness of the peaks and the breadth of summed activity of all the neurons that respond to the different frequency steps. This suggests that tuning curves, at least at the level of the inferior colliculus, are much sharper (have a higher Q-value) in *N. albiventris* than in *E. fuscus*. This is likely to be the case near the CF, although specialization for precise analysis of frequencies around the emitted CF in short CF-FM bats (Grinnell 1970; Grinnell and Hagiwara 1972; Wenstrup 1984) appears to be much less than in long CF-CM bats (Long and Schnitzler 1975; Suga and Jen 1977; Pollak et al. 1986). In any case, most of the FM sweep in *N. albiventris* would be below the frequency of specialized frequency resolution, and the requirement for 95–100 separate frequency filters is the same with 200–400 Hz steps as with 100 Hz steps.

The required step number and 100-Hz minimal step size in *N. albiventris* are consistent with the observation that interference with distance discrimination can be accomplished by a 10/s free-running artificial CF-FM sound with an FM bandwidth of 11 kHz or more (75–64 kHz) (Roverud and Grinnell 1985b). If one accepts 100 Hz as the size of a discriminable single step/frequency filter in *N. albiventris*, an FM sweep of 20 kHz corresponds to 200 separate frequency channels or filters, each reporting the same echo delay. In *E. fuscus*, 83 discriminable steps of 450 Hz would represent an FM sweep of ~ 37 kHz. In both species, the normal FM sweeps cover a larger range, which represents a significant safety margin for redundant measurement of echo delay. Presumably, the greater the redundancy, the more accurate the time measurement. Evidence for this can be seen in the finding that the microsecond precision and stability of evoked potentials in the inferior colliculus of *E. fuscus* increases with increasing FM bandwidth and sweep rate (Luo et al. 2018).

On the other hand, the experimentally derived estimate of safety margin in number of bandpass filters is surely a significant underestimate. Frequency resolution in the auditory system (the Weber fraction) is inversely proportional to the carrier frequency (i.e. the ratio of the just discriminable frequency divided by carrier frequency). Consequently, resolution of pure tone steps around 75 kHz requires a larger absolute frequency difference than at lower frequencies (Simmons et al. 2004). Given the requirement of ~ 95 separate steps in *N. albiventris*, 100-Hz steps must be resolvable as separate steps only at the highest frequencies. Progressively smaller steps would suffice at lower frequencies. This interpretation would help explain the relative importance of lower frequencies in echoes in supporting accurate distance measurement (Surlykke 1992; Bates and Simmons 2010). The ratio of minimum step size to carrier frequency at 75 kHz is 0.0013. If the same resolving power applies at lower frequencies of the sweep, the discriminable step size would be ~ 65 Hz at 50 kHz.

By the same reasoning, the minimum step size of 450 Hz in *E. fuscus* presumably applies only to the highest frequencies in the artificial 75–25 kHz pure tone FM sweep (ratio 0.006 at 75 kHz), and a requirement for 83 or more steps probably underestimates the number of discriminable narrow frequency filters. However, the requirement for 83 or more steps may explain the inability of *E. fuscus* to discriminate distance based on the second harmonic alone (Bates and Simmons 2010). With a significant second harmonic sweep length of 30 kHz (85–55 kHz), a frequency resolution of 450 Hz would provide only ~ 67 discriminable bandpass channels. If the ratio for larger steps above 75 kHz must be maintained at ~ 0.006, the number of discriminable steps in the second harmonic would be smaller still. In the context of echoes with both harmonics in register, however, the second harmonic could contribute many narrow frequency filter measurements of echo delay. The required FM sweep range equivalent to 83 or more steps of 450 Hz in *Eptesicus* is easily met in normal emitted approach phase pulses, which sweep through 50 kHz or more. However, search phase pulses, which are optimized for detection with much shallower FM sweeps often ending in a quasi-CF component, might not provide useful distance information.

The other major difference in the properties of the narrow frequency-tuned “filters” in the two species is in the more limited acceptable step duration: up to 80 µs in *N. albiventris* and only up to 50 µs in *Eptesicus*. This appears to fit the steeper sweep rate and wider bandwidth of *Eptesicus* approach phases pulses. On the other hand, the long shallow FM sweeps in *Eptesicus* search phase pulses may exceed this apparent 50-µs maximum duration in the range of any single frequency filter. The significantly longer (80 µs) maximum effective step duration in *N. albiventris* implies adaptation for more shallow FM sweeps. Nonetheless, the auditory nervous system may be internally programmable to alter the step size or duration restrictions derived from Roverud’s experiments. The fact that the artificial CF-FM pulses do not interfere with target detection or left–right direction discrimination (Roverud and Grinnell 1985a) suggest that these tasks, at least in their simplest form, do not face the same requirements as distance determination. It is quite possible –-even likely–-that a different number of pure tone steps would be required for more precise target localization or for characterization of target dimensions.

A remaining question is what is significant about 90–100 separate frequency filters in *N. albiventris* and 83 in *E. fuscus*? Are these the minimum number of frequency channels for an FM sweep to be misidentified as a true echo, or are they the minimum number of separate frequency channels, each reporting the same delay after an emitted cry, needed to provide a distance measurement? And what is the circuitry that integrates the activity of at least this number of separate frequency filters and makes the decision about target distance? *E. fuscus* forages for insect prey in open air; *N. albiventris* exhibits more flexible feeding behavior, typically hunting for insects above water, often pursuing and using their hypertrophied hind feet and claws to pick them off the water surface when they dive down to evade capture. They also catch small fish and hunt over land in more cluttered environments (Kalko et al. 1998). What these or other differences in echolocation behavior might help explain the differences described in this paper are questions for further thought.

## Conclusions

Novel behavioral experiments have revealed interesting properties of the neural circuitry that enables accurate distance discrimination and clutter exclusion in echolocating bats. In the short CF-FM bat *Noctilio albiventris,* the CF component of the emitted pulse gates a 20–30 ms period during which the auditory nervous system measures pulse echo delay at all the frequencies in the FM sweep. Echoes or FM signals outside of this time window are treated as not carrying time information but artificial sounds containing both the CF and FM components activate the same circuitry and interfere with time/distance measurement. The CF activation of the pulse-echo delay measurement circuitry has the interesting properties that the beginning of the CF must precede the FM sweep by 2 ms or more and the sweep must start at the same frequency as the CF. These are clues to the neural mechanisms involved that call for further studies. This role of the CF in *N. albivenris* may represent an unappreciated function of the CF component in the emitted orientation sounds of other (or all) short CF-FM bats and possibly in long CF bats as well, where multiple other functions are already clear.

Experiments in which the smooth FM sweep is replaced by a sequence of pure tone steps not only allow quantification of the minimum number of tuned filters in the filterbank array needed for distance determination, but they also illuminate some of the properties of the individual bandpass tuned filters, especially the minimum separation in frequency tuning between filters (step size) and in the maximum duration of the FM sweep within the bandwidth of individual frequency filters, setting a lower limit to sweep rate. The differences in these properties between *N. albiventris* and *E. fuscus* reflect differences in echolocation adaptations that are likely tailored to their different echolocation strategies. and are interesting clues for further research.

## Data Availability

No datasets were generated or analysed during the current study.
